# Uncovering the Binding Specificities of Lectins with Cells for Precision Colorectal Cancer Diagnosis Based on Multimodal Imaging

**DOI:** 10.1002/advs.201800214

**Published:** 2018-04-19

**Authors:** Rongrong Tian, Hua Zhang, Hongda Chen, Guifeng Liu, Zhenxin Wang

**Affiliations:** ^1^ State Key Laboratory of Electroanalytical Chemistry Changchun Institute of Applied Chemistry Chinese Academy of Sciences Changchun 130022 P. R. China; ^2^ School of Applied Chemistry and Engineering University of Science and Technology of China Road Baohe District Hefei Anhui 230026 P. R. China; ^3^ Department of Radiology China–Japan Union Hospital of Jilin University No. 126, Xiantai Street Changchun 130033 P. R. China

**Keywords:** colorectal cancer, lectin microarrays, tumor biomarkers, Uelx Europaeus Agglutinin‐I

## Abstract

There is a high desire for novel targets/biomarkers to diagnose and treat colorectal cancer (CRC). Here, an approach starting from a polyacrylamide hydrogel–based lectin microarray is presented to screen the high expression of glycans on the CRC cell surface and to identify new lectin biomarkers for CRC. Three common CRC cell lines (SW480, SW620, and HCT116) and one normal colon cell line (NCM460) are profiled on the microarray with 27 lectins. The experimental results reveal that CRC cells highly express the glycans with d‐galactose, d‐glucose, and/or sialic acid residues, and Uelx Europaeus Agglutinin‐I (UEA‐I) exhibits reasonable specificity with SW480 cells. After conjugation of UEA‐I with silica‐coated NaGdF_4_:Yb^3+^, Er^3+^@NaGdF_4_ upconversion nanoparticles, the follow‐up in vitro and in vivo experiments provide further evidence on that UEA‐I can serve as tumor‐targeting molecule to diagnose SW480 tumor by multimodal imaging including upconversion luminescence imaging, *T*_1_‐weighted magnetic resonance imaging, and X‐ray computed tomography imaging.

## 1 Introduction

Colorectal cancer (CRC) is one of the commonest alimentary system malignancy, which has relatively high incidence and mortality.^1^, ^2^, ^3^ Because of the commonality of malignancies including metastasis and recurrence, successful CRC therapy is strongly dependent on the detection of tumor at an earlier stage (early diagnosis), the correct identification of the subtype of cancer (precision treatment) and an accurate prediction of its likely course after suitable treatment (prognosis). For instance, the five year survival rate of patients with advanced CRC is significantly lower than that of patients with CRC at an early stage. Therefore, there is an urgent demand for novel biomarkers to diagnose CRC with high accuracy and sensitivity.

Glycans such as glycolipids and glycoproteins involve in many physiological processes including cell differentiation and proliferation, cell–cell communication, immune response, and tumor growth and metastasis.^4^, ^5^, ^6^, ^7^ Numerous cancers including CRC have been known to relate with structural abnormalities of N‐ and/or O‐linked glycoproteins.^8^, ^9^ Several glycoproteins are reported as candidate biomarkers for cancers, and a part of them have been approved for clinical diagnosis of cancers by the US Food and Drug Administration.^10^, ^11^ Currently, various methods/assays have been developed for glycan detection both on cell surface and in body fluids, such as high performance liquid chromatography, capillary electrophoresis, mass spectrometry (MS), enzyme‐linked immunosorbent assay, and microarrays.^12^, ^13^, ^14^, ^15^ Lectins, the carbohydrate binding proteins, exhibit high specificities for saccharide moieties, and can perform specific glycan recognition on the cellular and molecular level. Like antibodies, some lectins are known to play important roles in the immune system including defense against invading microorganisms and modulation of inflammatory and autoreactive processes through specific recognition of cellular and/or bacterial surface saccharide moieties. These characteristics make lectins very useful for glycoanalysis and medicine development.^16^, ^17^, ^18^, ^19^, ^20^, ^21^ Due to their minimization of sample consumption and high throughput format for analyzing multiple targets simultaneously, lectin microarrays have been employed in the glycomics study.^22^, ^23^, ^24^, ^25^, ^26^ However, the interactions of lectins with glycans (dissociation constant, *K*_d_ = 10^−4^–10^−7^ m) are much weaker than the interactions of antigens with antibodies (*K*_d_ = 10^−8^–10^−12^ m). The cells are easily washed off from lectin microarray during the subsequent cleaning and drying process since the binding affinity between a single glycan molecule and lectin on a planar surface is relatively low, leading to reducing of the accuracy and reproducibility of analysis results. The drawback limits the application of conventional 2D lectin microarray for directly profiling glycan expression on cell surface. Comparing with 2D planar substrates, 3D substrates have many advantages including high probe loading capacity and optimal reaction space for adjusting biomolecular distribution.^27^, ^28^, ^29^, ^30^, ^31^ In particular, the carbohydrate–lectin interactions can be strengthened on the 3D microarray through formation of multivalent binding among the immobilized probe molecules. However, there are a few examples of 3D lectin microarrays for profiling cellular glycan expression and screening lectin biomarker.

Multimodal imaging has many advantages such as improving diagnostics or guidance through the analysis of complementary, data‐rich, coregistered images to address weaknesses in individual imaging modalities.^32^, ^33^ Because of their unique upconversion luminescence (UCL) and strong contrast enhancement of magnetic resonance imaging (MRI) and X‐ray computed tomography (CT) imaging, NaGdF_4_‐based upconversion nanoparticles (UCNPs) have been demonstrated as one of the most appealing contrast agents for multimodal imaging (UCL/MRI/CT).^34^, ^35^, ^36^, ^37^, ^38^ Due to undesired accumulation of nanoparticles (NPs) in the liver, spleen, and kidneys, only a small proportion (less 10% of the injected dose (ID) g^−1^) of passive tumor‐targeting NPs can reach the tumor sites through the enhanced permeability and retention effect.^35^, ^39^ The tumor accumulation efficiency can be improved through conjugation of NPs with tumor‐targeting ligand which can specifically bind with the receptor overexpressed on the membrane of tumor cell or tumor vasculature cell.^40^, ^41^, ^42^ Because aberrant glycan patterns have already been considered as a hallmark of cancers, lectins may serve as tumor‐targeting agents through multivalent binding of glycans on the cell surface. For example, concanavalin A‐modified UCNPs have been successfully used to label cell surface glycan labeling and differentiate between HCCHM3 and CL cells.^43^

In present study, a polyacrylamide (PAAM) hydrogel–based lectin microarray has been fabricated for profiling glycan expression on CRC cell surface and screening new lectin biomarkers for CRC. The immobilized lectins on PAAM hydrogel may serve as multivalent binding scaffolds to the cellular glycans, resulting in increased binding affinity and selectivity. Uelx Europaeus Agglutinin I (UEA‐I) has been demonstrated to have high affinity and specificity with SW480 cells through the interactions of 27 different lectin species with 4 distinct cell types. Using silica‐coated NaGdF_4_:Yb^3+^, Er^3+^@NaGdF_4_ UCNP as nanoprobe, the UEA‐I has been successfully applied to differentiate SW480 cells and mouse‐bearing SW480 tumor by multimodal imaging including UCL imaging, *T*_1_‐weighted MRI, and CT imaging.

## 2 Results and Discussion

### 2.1 PAAM Hydrogel–Based Lectin Microarray Fabrication

**Figure 1:**
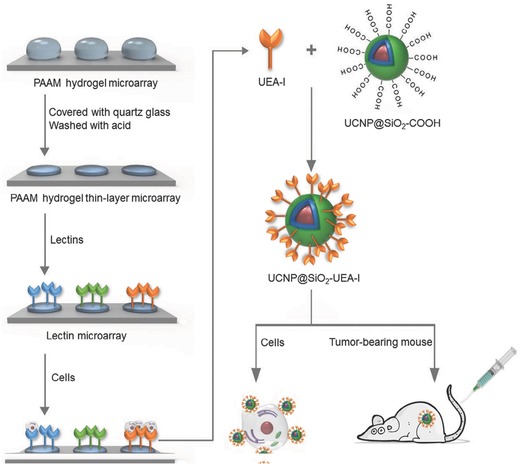
Schematic representation of evaluation of lectin biomarker for CRC.

The PAAM hydrogel microarray was prepared by our previously reported method with slight modifications (as shown in **Figure** **1**).^44^ The morphology of PAAM hydrogel spot was characterized by X‐ray photoelectron spectroscopy (XPS), scanning electron microscopy (SEM), and atomic force microscopy (AFM). After gelation, the N1s peak has been clearly observed on the surface of PAAM hydrogel spot (as shown in Figure S1 in the Supporting Information).^45^, ^46^ The result demonstrates that the PAAM hydrogel microarray has been successfully fabricated. Representative SEM and AFM images reveal that the PAAM hydrogel spot has a relatively rough surface and the size of PAAM hydrogel spot is about 500 µm in diameter (as shown in Figures S2 and S3 in the Supporting Information). The results indicate that the PAAM hydrogel spot can provide relatively large surface area for immobilization of lectin molecules. The carboxyl groups of PAAM hydrogel were activated by traditional 1‐ethyl‐3‐(3‐dimethylaminopropyl) carbodiimide hydrochloride (EDC)/*N*‐hydroxysulfosuccinimide sodium salt (sulfo‐NHS) reaction, which leads to the formation of an outer layer of NHS ester group on PAAM hydrogel. The PAAM hydrogel–based lectin microarray was achieved by noncontact spraying lectin droplets to activate PAAM hydrogel microarray. The amino residues of lectin can react with NHS ester group to form stable amide bond.

The interactions of HeLa cells with three lectins (lens culinaris agglutinin (LCA), ricinus communis agglutinin I (RCA 120), and pisum sativum agglutinin (PSA)) have been arbitrarily selected to evaluate the binding performance of PAAM hydrogel–based lectin microarray with living cells since HeLa cells have high affinities with these lectins.^47^, ^48^, ^49^ As shown in Figure S4 (Supporting Information), the relative high signal intensities can be achieved with 1 mg mL^−1^ lectins in spotting solution and 1 × 10^6^ cells mL^−1^ HeLa cells in cell buffer. The high signal‐to‐background ratio (*S*/*N*) is obtained with incubation of HeLa cells with lectins microarray for 40 min. Taking into account the sensitivity and specificity of the cell–lectin interaction, the following experiments were carried out under the optimized conditions: 1 mg mL^−1^ lectins in spotting solution, 1 × 10^6^ cells mL^−1^ cells in cell buffer, and 40 min for incubation.

### 2.2 Screening Specific Lectin for CRC Cells

**Figure 2:**
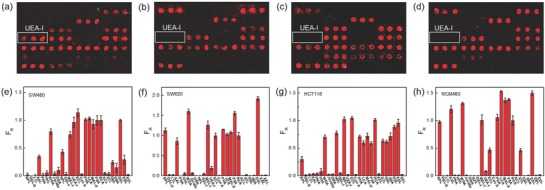
The fluorescence images and corresponding data analysis of the interactions of 27 lectins with a,e) SW480, b,f) SW620, c,g) HCT116, and d,h) NCM460 cells, respectively. The lectin arrangement is shown in Table S3 (Supporting Information). *F*_R_ represents the relative fluorescence intensity. The error bars mean standard deviations from three duplicated lectin spots.

To screen specific binding of lectin with CRC, we examined the interactions of 27 lectins with 4 cell lines including three CRC cell lines (SW480, SW620, and HCT116) and one normal colon cell line (NCM460). The three CRC cell lines are derived from three kinds of common CRC. Generally, SW480 and HCT116 cells are derived from the stage 2 primary CRC and stage 4 primary CRC, respectively, while SW620 cells are derived from the stage 3 CRC with lymph node metastasis.^50^ Because RCA 120 has strong binding affinity with almost all mammalian cells, the binding of RCA 120 with cells is used as the internal control in this study. After incubation with selected cells, the fluorescence intensities of lectin–cell pairs (the lectin arrangement is shown in Table S3 in the Supporting Information) were recorded. Then, the relative fluorescence intensity (*F*_R_ = (*F*_Lectin_ − *F*_0_)/(*F*_RCA 120_ − *F*_0_)) was calculated and employed to evaluate the binding affinity of lectin with living cells, where *F*_Lectin_, *F*_RCA 120_, and *F*_0_ are the average fluorescence intensity of a certain lectin–cell pair, the average fluorescence intensity of RCA 120–cell pair, and the average fluorescence intensity of control, respectively. The fluorescence images of microarray and the corresponding *F*_R_ are illustrated in **Figure** **2**. The binding affinities of lectins with cells were divided into 4 grades, i.e., strong binding (*F*_R_ ≥ 0.8, indicated as: +++), medium binding (0.5 ≤ *F*_R_ < 0.8, indicated as: ++), weak binding (0.2 ≤ *F*_R_ < 0.5, indicated as: +), and nonbinding (*F*_R_ < 0.2, indicated as: ‐), which were summarized in **Table** **1**. As expected, all of these cells have reasonable binding affinities with RCA 120, which suggests that the cells express high level of d‐galactose (Gal) residues in their surface glycans. This phenomenon is consistent with previous studies.^51^, ^52^, ^53^ All 4 cell lines herein have strongly binding behaviors with 9 lectins including wheat germ agglutinin (WGA), maackia amurensis lectin I (MAL I), erythrina cristagalli lectin (ECL), phaseolus vulgaris agglutinin (PHA‐E+L), LCA, datura stramonium lectin (DSL), maackia amurensis lectin II (MAL II), PSA, and RCA 120, which indicates that the carbohydrate residues on these cellular surfaces have partial consistency. For instance, these cells may express high levels of Gal, d‐glucose, and/or sialic acid residues on their cellular surfaces. The difference in the lectin binding patterns of CRC cells and the normal colon cells has also been demonstrated, suggesting differences in carbohydrate motifs on the cellular surfaces. For example, three lectins (narcissus pseudonarcissus (daffodil) lectin (NPL), galanthus nivalis lectin (GNL), and elderberry bark lectin (EBL)) exhibit reasonable binding affinities with SW480 cells and HCT116 cells, while these lectins have poor binding affinities with SW620 cells and NCM460 cells. The result indicates that SW480 cells and HCT116 cells strongly express mannose residue complexes and/or Neu5Acα6Gal glycosyl complexes. In particular, only SW480 cells show strong binding affinity with UEA‐I (as shown in Figure S5 in the Supporting Information), suggesting that SW480 cells express high level of α‐1,2‐fucosylation complexes on the surface.^54^ This phenomenon is consistent with the high expression level of H α‐2‐fucosyltransferase messenger RNA (mRNA) in SW480 cells.^55^ Therefore, UEA‐I could be used as a lectin biomarker to discriminate SW480 CRC subtype.

**Table 1:** The summary of interactions of 27 different lectin species with 4 distinct cells

Lectina)	SW480	SW620	HCT116	NCM460	Lectin	SW480	SW620	HCT116	NCM460
Bauhinia Purpurea Lectin (BPL)	‐	+++	+	+++	Concanavalin (Con A), Unconjugated	‐	‐	‐	‐
Griffonia simplicifolia lectin II (GSL II)	‐	‐	‐	‐	PHA‐E+L	+++	+++	++	+++
Lotus tetragonolobus lectin (LTL)	‐	‐	‐	‐	LCA	+++	+++	++	+++
Aleuria aurantia lectin (AAL)	+	+++	‐	+++	MAL‐II	+++	+++	++	+++
Peanut agglutinin (PNA)	‐	‐	‐	‐	PSA	+++	+++	++	+++
Hippeastrum hybid (Amaryllis) lectin (HHL)	‐	‐	‐	‐	RCA 120	+++	+++	+++	+++
WGA	++	+++	++	+++	Amaranthus caudatus lectin (ACL)	‐	‐	‐	‐
Jacalin	‐	‐	‐	‐	Solanum tuberosum (Potato) lectin (STL)	‐	+	++	+
Dolichos biflorus agglutinin (DBA)	‐	‐	‐	‐	GNL	+	‐	++	‐
NPL	++	‐	++	‐	Soybean agglutinin (SBA)	‐	‐	++	‐
Griffonia simplicifolia lectin I (GSL I)	‐	‐	‐	‐	DSL	+++	+++	+++	+++
MAL‐I	++	+++	+++	+++	EBL	+	‐	+++	‐
UEA‐I	+++	‐	‐	‐	Euonymus europaeus lectin (EEL)	‐	‐	‐	‐
ECL	+++	+++	+++	++					

^a)^ *F*_R_ ≥ 0.8: +++; 0.5 ≤ *F*_R_ < 0.8: ++; 0.2 ≤ *F*_R_ < 0.5: +; *F*_R_ < 0.2: ‐.

### 2.3 Preparation and Characterization of UCNP@SiO_2_–UEA‐I

To verify its capability, the UEA‐I was covalently conjugated with UCNP@SiO_2_—COOH through reactions of the carboxyl groups on the UCNP surfaces with the primary and secondary amines residues of UEA‐I.^56^ In this case, UCNP@SiO_2_—COOH was used as nanoprobe because that carboxyl‐terminated SiO_2_ shell has weak nonspecific binding with cellular membrane and NaGdF_4_:Yb^3+^, Er^3+^@NaGdF_4_ UCNP can serve as multifunction contrast agent for multimodal imaging (UCL/MRI/CT). UCNP@SiO_2_—COOH was synthesized by previously reported strategy.^57^, ^58^ The UCL emission of NaGdF_4_:Yb^3+^, Er^3+^@NaGdF_4_ is much stronger than that of NaGdF_4_:Yb^3+^, Er^3+^ UCNPs since the outer NaGdF_4_ shell can protect the migrating energy in Gd sublattice from trapping by surface quenchers.^59^ The average size of UCNP@SiO_2_—COOH is 21.0 ± 0.5 nm in diameter including a NaGdF_4_:Yb^3+^, Er^3+^ core with the size of 10.0 ± 0.5 nm in diameter, a NaGdF_4_ inner shell with the thickness of ≈2.5 nm and a SiO_2_ outer shell with the thickness of ≈3 nm (as shown in Figure S6 in the Supporting Information). After UEA‐I conjugation, the morphology, dispersity, and UCL emission of UCNP@SiO_2_—COOH exhibit a negligible change (as shown in Figure S6 in the Supporting Information). The corresponding X‐ray diffraction patterns (as shown in Figure S7 in the Supporting Information) of NaGdF_4_:Yb^3+^, Er^3+^ UCNPs and NaGdF_4_:Yb^3+^, Er^3+^@NaGdF_4_ UCNPs indicate that they are pure hexagonal phase (JCPDS No. 27‐0699). The XPS measurements clearly show the element of Si in UCNP@SiO_2_—COOH and the elements of N and Si in UCNP@SiO_2_–UEA‐I (as show in Figure S8 in the Supporting Information). The Fourier transform infrared (FTIR) spectrum of UCNP@SiO_2_–UEA‐I exhibits one peak at 1639 cm^−1^, which is associated with stretching vibration of amide bonds (as shown in Figure S9 in the Supporting Information). The XPS and FTIR results confirm that UEA‐I has been successfully conjugated on UCNP@SiO_2_—COOH surface. After conjugation of UEA‐I, the hydrodynamic diameter and zeta potential of UCNP@SiO_2_—COOH are changed from 29.4 ± 1.1 to 108.1 ± 2.0 nm, and ‐43.7 ± 0.5 to −10.5 ± 1.0 mV, respectively, which gives further evidence on successful preparation of UCNP@SiO_2_–UEA‐I (as shown in Figure S10 in the Supporting Information).

To evaluate their contrast enhancement capacities for MR/CT imaging, *T*_1_‐weighted MR and CT imaging of UCNP@SiO_2_—COOH and UCNP@SiO_2_–UEA‐I were investigated. Both MR and CT signals are linearly increased with increasing the concentrations of UCNP@SiO_2_—COOH and UCNP@SiO_2_–UEA‐I (as shown in Figures S11 and S12 in the Supporting Information). The molar longitudinal relaxivities (*r*_1_ = 1/*T*_1_, the slopes of lines in Figure S12 in the Supporting Information) of UCNP@SiO_2_—COOH and UCNP@SiO_2_–UEA‐I were calculated to be 7.05 and 8.73 mm ^−1^ s^−1^, respectively. The higher *r*_1_ value of UCNP@SiO_2_–UEA‐I might be due to the polar amino acids in UEA‐I and the rigidity of UEA‐I, which increase the density of water molecule around gadolinium ions through accelerating the exchange rate of intraspherical water molecule.^60^, ^61^ The hounsfield unit (HU) values (200) of 4.3 mg mL^−1^ UCNP@SiO_2_—COOH and UCNP@SiO_2_–UEA‐I are equal to that of 8.0 mg mL^−1^ iodine in Omnipaque (a contrast agent for clinical CT imaging), indicating that these NPs have high CT contrast enhancement ability.

In addition, the SW480 cells still have more than 80% viability after incubated with as high as 200 µg mL^−1^ NPs for 24 h (as shown in Figure S13 in the Supporting Information), suggesting that both of UCNP@SiO_2_—COOH and UCNP@SiO_2_–UEA‐I exhibit low cytotoxicity.

### 2.4 Interactions of UCNP@SiO_2_–UEA‐I with Cells

**Figure 3:**
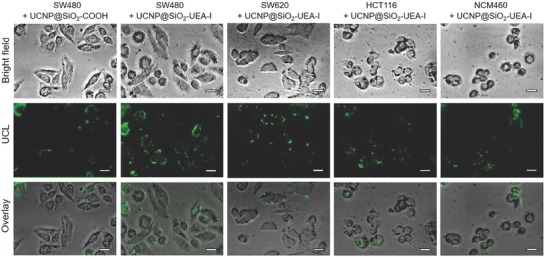
UCL images of SW480 cells, SW620 cells, HCT116 cells, and NCM460 cells treated with UCNP@SiO_2_–UEA‐I (100 µg mL^−1^), and SW480 cells treated with UCNP@SiO_2_—COOH (100 µg mL^−1^) for 0.5 h, respectively. Scale bars are 20 µm.

As shown in **Figure** **3**, the UCNP@SiO_2_–UEA‐I‐stained SW480 cells exhibit the highest fluorescence signal in all of the NP‐stained cells. The maximum UCL intensity at 540 nm of UCNP@SiO_2_–UEA‐I‐stained SW480 cells is 2.1‐fold of UCNP@SiO_2_–UEA‐I‐stained SW620 cells, 3.4‐fold of UCNP@SiO_2_–UEA‐I‐stained HCT116 cells, 3.2‐fold of UCNP@SiO_2_–UEA‐I‐stained NCM460 cells, and 3.8‐fold UCNP@SiO_2_—COOH‐stained SW480 cells, respectively (as shown in Figure S14 in the Supporting Information). In addition, the MR and CT signal intensities of UCNP@SiO_2_–UEA‐I‐stained SW480 cells are much higher than those of UCNP@SiO_2_–UEA‐I‐stained other cells and that of UCNP@SiO_2_—COOH‐stained SW480 cells (as shown in Figure S15 in the Supporting Information). The results confirm that UEA‐I has high affinity with SW480 cells.

### 2.5 SW480 Tumor‐Targeting Ability of UCNP@SiO_2_–UEA‐I

**Figure 4:**
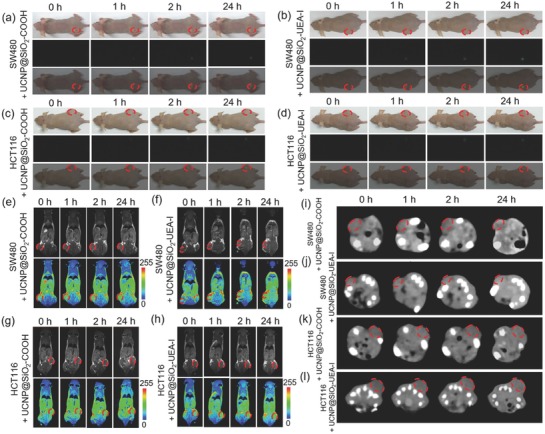
In vivo a–d) UCL (from up to bottom: bright field mode, UCL mode (green channel), and merging mode), e–h) MR, and i–l) coronal CT images of SW480 tumor‐ and HCT116 tumor‐bearing nude mice after intravenous injection of UCNP@SiO_2_—COOH and UCNP@SiO_2_–UEA‐I at different timed intervals, respectively (the 0 h means preinjection).

**Figure 5:**
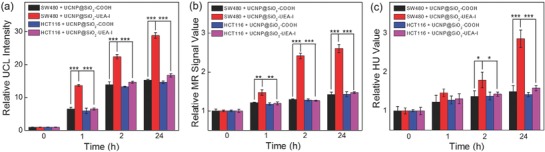
In vivo relative a) UCL, b) MR, and c) CT signals of SW480 tumor‐ and HCT116 tumor‐bearing nude mice after intravenous injection of UCNP@SiO_2_—COOH and UCNP@SiO_2_–UEA‐I at different timed intervals, respectively (the 0 h means preinjection). The error bars are standard deviations (*n* = 3. The significance of data is analyzed according to one‐sided paired Student's *t*‐test: *p < 0.05, **p < 0.01, and ***p < 0.001).

**Figure 6:**
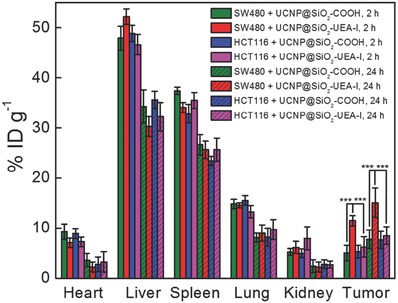
Biodistribution of Gd element in tumor and main organs of mice at 2 and 24 h post‐intravenous injection of UCNP@SiO_2_—COOH and UCNP@SiO_2_–UEA‐I (0.3 mg in 200 µL NaCl solutions (0.9 wt%)), respectively. The error bars are standard deviations (*n* = 3. The significance of data is analyzed according to one‐sided paired Student's *t*‐test: ***p < 0.001).

The BALB/c mice bearing xenograft SW480 tumor model and/or HCT116 tumor model were established for testing the SW480 tumor‐targeting ability of UCNP@SiO_2_–UEA‐I by in vivo multimodal imaging (UCL/MRI/CT) evaluation. The UCNP@SiO_2_–UEA‐I or UCNP@SiO_2_—COOH were intravenously injected into the mice through tail veins. The whole body UCL/MR/CT images of mice were recorded at the desired time points of postinjection. For all the cases, significant UCL/MR/CT contrast enhancement in the tumor sites is observed after 1 h postinjection of NPs (as shown in **Figures** **4** and **5** and Tables S3 and S4 (Supporting Information)). In particular, the UCL, MR, and CT contrast enhancements of SW480 tumor sites with UCNP@SiO_2_–UEA‐I are much stronger than those of SW480 tumor sites with UCNP@SiO_2_—COOH, HCT116 tumor sites with UCNP@SiO_2_–UEA‐I, and HCT116 tumor sites with UCNP@SiO_2_—COOH. The experimental result demonstrates that the as‐prepared UCNP@SiO_2_–UEA‐I has good SW480 tumor‐targeting ability. Moreover, the tumor and main organs including heart, liver, spleen, lung, and kidneys were collected at 2 and 24 h postinjection of NPs. The amounts of Gd element in these tissues were then analyzed by inductively coupled plasma mass spectrometry (ICP‐MS) (as shown in **Figure** **6**). After 24 h injection, the accumulation amount of UCNP@SiO_2_–UEA‐I in SW480 tumor (15.0% ID g^−1^) is much higher than those of UCNP@SiO_2_—COOH in SW480 tumor (7.8% ID g^−1^), UCNP@SiO_2_–UEA‐I in HCT116 tumor (8.5% ID g^−1^), and UCNP@SiO_2_—COOH in HCT116 tumor (7.6% ID g^−1^). These experimental results clearly demonstrate that the UEA‐I can serve as a biomarker for SW480 tumor diagnosis through specific binding with α‐1,2‐fucosylation glycan on the cellular surface.

### 2.6 In Vivo Toxicology Investigation

The histochemical analysis indicates that there are no notable evidences of tissue damage and adverse effect of the UCNP@SiO_2_–UEA‐I to major organs of BALB/c mice including heart, liver, spleen, lung, and kidneys at 30 d postinjection (as shown in Figure S16 in the Supporting Information). Comparison with the control group, UCNP@SiO_2_–UEA‐I‐treated mice exhibit negligible difference on the numbers of blood cells (as shown in Table S3 in the Supporting Information). The results confirm the low toxicity of UCNP@SiO_2_–UEA‐I.

## 3 Conclusion

In summary, the present study provides a proof‐of‐principle demonstration of the screening candidate lectin biomarkers for CRC through direct profiling of binding affinities of living cells with lectins on PAAM hydrogel microarray. UEA‐I is identified to have specific binding affinity with SW480 cells by interactions of four kinds of colorectal organ/tumor–derived living cells with 27 lectins. After conjugation of UEA‐I with UCNP@SiO_2_—COOH, the UCNP@SiO_2_–UEA‐I provides high specificity toward mouse‐bearing SW480 tumor, and displays reasonable tumor contrasts as shown in multimodal imaging (UCL/MRI/CT) studies. This exemplifies its potential for noninvasive diagnosis of CRC at subtype level. Although only limited interactions of cells with lectins have been studied, the finding would open up possibilities for the future to discover lectins as biomarkers toward a broader biomedical application including cancer diagnosis and therapy.

## 4 Experimental Section

*Fabrication of the Polyacrylamide Hydrogel Microarray*: The PAAM hydrogel microarray was prepared by the previously reported method with slight modifications.^41^ Using the SmartArray‐136 system (CapitalBio Ltd., China), the gel precursor aqueous solutions (3% w/v acrylamide, 0.12% w/v bis‐acrylamide, 10% w/v acrylic acid, and 15% v/v glycerol in water) were spotted on the benzophenone (BP)‐treated glass slides at the desired array by noncontact spraying, then each glass slide was covered with a quartz slide (125 mm × 40 mm × 1 mm) and irradiated by UV light (λ = 365 nm, light intensity = 4 mW cm^−2^) for 4 min. After photopolymerization, the slides were immersed in phosphate buffer (PB) solution (pH 5.7, 100 × 10^−3^ m NaH_2_PO_4_·2H_2_O, and 15 × 10^−3^ m Na_2_HPO_4_·12H_2_O) and shaken for 30 min at room temperature. After thoroughly washed by water (30 mL, 3 times), the PAAM hydrogel microarrays were activated by 1 mL of EDC and sulfo‐NHS solution (50 × 10^−3^ m in 4‐morpholineethanesulfonic acid hydrate (MES) buffer, pH 5.7) for 1 h, followed by washing with 30 mL MES buffer (2 times) and 30 mL water (1 time), respectively. After dried by centrifugation (200 × *g* for 1 min), the activated PAAM hydrogel microarrays were stored at 4 °C.

*Fabrication of Lectin Microarrays*: To fabricate lectin microarrays, lectins were dissolved in spotting buffer (pH 8.0, 10 × 10^−3^ m *N*‐(2‐hydroxyethyl)piperazine‐29‐(2‐ethane‐sulfonic acid), 0.15 m NaCl, 0.02% v/v Tween‐20, 0.005% w/v bovine serum albumin (BSA), and 30% v/v glycerol) at the desired concentration and sprayed on the PAAM hydrogel spots by SmartArray‐136 microarrayer with noncontacting spraying, respectively. After incubation under 60% humidity at 25 °C for 12 h, the lectin microarrays were washed with 30 mL washing buffer (pH 7.5, 50 × 10^−3^ m PB, 0.15 m NaCl, 0.2% w/v BSA, and 0.1% v/v Tween‐20, 3 times), incubated with 30 mL blocking buffer (pH 7.5, 50 × 10^−3^ m PB, 0.15 m NaCl, 0.5% w/v BSA, and 1% v/v ethanomine) at 25 °C for 1 h. After washed with washing buffer (30 mL, 3 times) and water (30 mL, 3 times), the lectin microarrays were dried by centrifugation (200 × *g* for 1 min) and stored at 4 °C.

*Screening Specific Lectin for CRC Cells*: Three common CRC cell lines (SW480, SW620, and HCT116), one normal colon cell line (NCM460) and one cervical carcinoma cell line (HeLa) were cultured in the desired fresh medium supplemented with 10% fetal bovine serum and 100 U mL^−1^ penicillin–streptomycin in humidified air with 5% CO_2_ at 37 °C. Leibovitz's L‐15 was used for culturing SW480 and SW620 cells, McCoy's 5A was used for culturing HCT116 and NCM460 cells, and Dulbecco's modified Eagle medium was used for culturing HeLa, respectively.

To screen specific binding of lectin with CRC cells, SW480, SW620, HCT116, and NCM460 were profiled on the microarray with 27 lectins. The layout of the lectin microarray with 27 lectins is shown in Table S2 (Supporting Information). After detached by trypsin, the living cells were collected by centrifugation and resuspended in cell buffer (pH 7.4, 1.5 × 10^−3^ m KH_2_PO_4_, 8 × 10^−3^ m Na_2_HPO_4_·12H_2_O, 137 × 10^−3^ m NaCl, 50 × 10^−6^ m CaCl_2_, and 50 × 10^−6^ m MnCl_2_), respectively. Then, the cells with various concentrations were incubated with lectin microarrays under 60% humidity in dark at room temperature for the desired time. After removing unbound cells by a pipette, the lectin microarrays were gently rinsed with 1 mL phosphate‐buffered saline (PBS) (pH 7.4 1.5 × 10^−3^ m KH_2_PO_4_, 8 × 10^−3^ m Na_2_HPO_4_·12H_2_O, and 137 × 10^−3^ m NaCl). Then, the cells were fixed by 4% paraformaldehyde for 20 min and labeled by propidine iodide (20 µg mL^−1^) in PBS for 10 min. Finally, the lectin microarrays were gently washed by submerging and inverting in 200 mL PBST (PBS with 0.5% Tween‐20), 200 mL PBS (7.4), and 200 mL water, respectively. After dried in air, the lectin microarrays were scanned by a LuxScan‐10K fluorescence microarray scanner (CapitalBio Ltd., China) with a red channel.

*Synthesis of the UCNP@SiO_2_*—*COOH*: The NaGdF_4_:Yb^3+^, Er^3+^ UCNPs were synthesized by a previously reported method.^54^ The carboxyl‐terminated silica‐coated NaGdF_4_:Yb^3+^, Er^3+^@NaGdF_4_ UCNPs (UCNP@SiO_2_—COOH) were synthesized by a reported water‐in‐oil microemulsion method with slightly modification.^55^ Typically, 1.8 mL TritonX‐100, 1.8 mL *n*‐hexanol, 0.48 mL deionized water, and 7.5 mL NaGdF_4_:Yb^3+^, Er^3+^@NaGdF_4_ in cyclohexane (6 mg mL^−1^) were mixed and stirred for 30 min. Then, 90 µL NH_3_·H_2_O (28 wt%) and 120 µL tetraethyl orthosilicate (TEOS) were added into the reaction mixture. After stirred for 5.75 h, 140 µL carboxyethylsilanetriol (CTES) was added and kept stirring at room temperature for another 24 h. The as‐prepared NaGdF_4_:Yb^3+^, Er^3+^@NaGdF_4_@SiO_2_—COOH NPs (termed as UCNP@SiO_2_—COOH) were precipitated by adding 5 mL acetone, collected by centrifugation, and washed with ethanol for several times. Finally, the UCNP@SiO_2_—COOH was redispersed in water.

*Synthesis of the UCNP@SiO_2_–UEA‐I*: 1 mL UCNP@SiO_2_—COOH (0.96 mg mL^−1^) in MES buffer (0.1 m, pH 6.0) was mixed with 0.28 mL EDC (2 mg mL^−1^) and 0.28 mL sulfo‐NHS (8 mg mL^−1^),^53^ and stirred at room temperature for 1 h. After centrifuged and washed with 1 mL MES buffer (0.1 m, pH 6.0), the precipitate was redispersed in 3 mL HEPES buffer (0.01 m, pH 7.2) containing 0.1 mg UEA‐I. The mixture was shaken (100 rpm) at 37 °C for 3 h. Finally, UEA‐I‐conjugated UCNP@SiO_2_ (termed as UCNP@SiO_2_–UEA‐I) was purified by repeated centrifugation (13 000 rpm, 20 min, 4 °C, 3 times) and resuspended in 0.5 mL PBS (pH 7.4).

*Cytotoxicity Measurement of UCNP@SiO_2_*—*COOH and UCNP@SiO_2_–UEA‐I*: The SW480 cells were cultured in 96‐well cell‐culture plate (1 × 10^4^ cells per well in 100 µL culture medium) under a humidified 5% CO_2_ at 37 °C for 24 h, followed by introduction of UCNP@SiO_2_—COOH and UCNP@SiO_2_–UEA‐I with the desired concentrations (6.25, 12.5, 25, 50, 100, and 200 µg mL^−1^) in 100 µL fresh culture medium, respectively. The cells were then incubated at the same conditions for 24 h, respectively. Cell viability was determined by traditional 3‐(4,5‐dimethylthiazol‐2‐yl)‐2,5‐diphenyltetrazolium bromide (MTT) assay.

*In Vitro UCL, MR, and CT Imaging*: For UCL imaging, SW480 cells, SW620 cells, HCT116 cells, and NCM460 cells (5 × 10^4^ cells per well in 0.5 mL culture medium) were seeded in 48‐well culture plates for 24 h. The culture medium was discharged and the cells were washed with PBS. Subsequently, 100 µg mL^−1^ UCNP@SiO_2_—COOH or UCNP@SiO_2_–UEA‐I in 0.5 mL fresh culture medium was introduced into each well and incubated at 37 °C for another 0.5 h, respectively. After washed with PBS (3 times), the NP‐stained cells were fixed with 4% paraformaldehyde for 20 min and subjected to UCL imaging under an external 980 nm laser (0.5 W cm^−2^). For CT and MR imaging, SW480 cells, SW620 cells, HCT116 cells, and NCM460 cells (1 × 10^6^ cells per well in 2.5 mL culture medium) were seeded in 6‐well culture plates for 24 h. The culture medium was discharged and the cells were washed with PBS. Then, 100 µg mL^−1^ UCNP@SiO_2_—COOH or UCNP@SiO_2_–UEA‐I in 2.5 mL fresh culture medium was introduced into each well and incubated at 37 °C for another 1 h, respectively. The cells were detached by 1 mL trypsin and centrifuged at 1000 rpm for 5 min. The supernatants were discharged. Subsequently, 1 × 10^6^ cells were immobilized in the 1.5 mL Eppendorf tubes by 1% agarose, respectively. The corresponding unstained SW480 cells were employed as control samples. *T*_1_‐weighted MR images were collected using a GE Signa 1.5‐T MR unit with the following imaging parameters: repetition time (TR), 240 ms; echo time (TE), 15.9 ms; field of view, 120 mm × 72 mm; slice thickness, 2.0 mm. CT imaging was performed as previously described.

*In Vivo UCL, MR, and CT Imaging*: BALB/c nude mice (six week old, 20 ± 0.2 g, male) were purchased from Beijing HFK Biotechnology Ltd. (Beijing, China). Animal experiments were conformed to the guidelines of the Regional Ethics Committee for Animal Experiments established by the Jilin University Institutional Animal Care and Use. The mice were subcutaneously inoculated with SW480 cells and HCT116 cells, respectively.

For in vivo multimodal (UCL/MR/CT) imaging, the SW480 tumor‐ and HCT116 tumor‐bearing nude mice were injected intravenously with NaCl solution (0.9 wt%, 200 µL) containing the desired amounts of UCNP@SiO_2_—COOH or UCNP@SiO_2_–UEA‐I (Gd^3+^: 1.5 mg mL^−1^ for UCL, 1.5 mg mL^−1^ MR imaging, and 15 mg mL^−1^ for CT imaging), respectively. The images were collected at the appropriate time points after injection. The in vivo UCL images were obtained by a CCD camera with a 980 nm NIR laser at the power density of 1.0 W cm^−2^ as excitation light. In vivo MR and CT images were collected as previously described, except a 129 mm field of view was used for CT.

*In Vivo Biodistribution and Toxicology Investigation*: For biodistribution study, the SW480 tumor‐ and HCT116 tumor‐bearing nude mice were sacrificed at 2 and 24 h postinjection of UCNP@SiO_2_—COOH or UCNP@SiO_2_–UEA‐I (200 µL, 1.5 mg mL^−1^ Gd^3+^), respectively. Then, the distribution of UCNP@SiO_2_—COOH or UCNP@SiO_2_–UEA‐I in tumors and main organs including heart, liver, spleen, lung, and kidney were digested in aqua regia under heat treatment (80 °C) for 2 h, and the as‐obtained liquids were subjected to ICP‐MS analysis. Histology analysis and blood biochemistry assay were employed to evaluate the biosafety of UCNP@SiO_2_–UEA‐I.

## Conflict of Interest

The authors declare no conflict of interest.

## Supporting information

Supplementary
