# Supplementary material for: Uncovering the Binding Specificities of Lectins with Cells for Precision Colorectal Cancer Diagnosis Based on Multimodal Imaging
Source: Adv Sci (Weinh). 2018 Apr 19;5(6):1800214. doi: 10.1002/advs.201800214 (PMC6010763; doi:10.1002/advs.201800214)
Supplement: Supplementary file 1 — Supplementary [file ADVS-5-1800214-s001.pdf]

Copyright WILEY-VCH Verlag GmbH & Co. KGaA, 69469 Weinheim, Germany, 2018.

ADVANCED SCIENCE Open Access

Supporting Information

for *Adv. Sci.,* DOI: 10.1002/advs.201800214

Uncovering the Binding Specificities of Lectins with Cells for Precision Colorectal Cancer Diagnosis Based on Multimodal Imaging

Rongrong Tian, Hua Zhang, Hongda Chen, Guifeng Liu, and Zhenxin Wang**

WILEY-VCH

Copyright WILEY-VCH Verlag GmbH & Co. KGaA, 69469 Weinheim, Germany, 2016.

Supporting Information

Uncover the Binding Specificities of Lectins with Cells for Precision Colorectal Cancer Diagnosis Based on Multimodal Imaging

Rongrong Tian, Hua Zhang, Hongda Chen, Guifeng Liu and Zhenxin Wang**

1. Additional Experimental Section

2. Additional Figures S1-S16

3. Additional Tables S1-S5

1. Additional Experimental Section

Material: Acrylamide (AAm, 40% (w/v) in water) and N-Hydroxysulfosuccinimide sodium salt (sulfo-NHS) were obtained from Aladdin Industrial Co. (Shanghai, China); 1-Ethyl-3-(3-dimethylaminopropyl) carbodiimide hydrochloride (EDC), rare-earth oxides including gadolinium oxide (Gd_2O_3, 99.99%), ytterbium oxide (Yb_2O_3, 99.99%) and erbium oxide (Er_2O_3, 99.99%), ammonium hydroxide (ACS, 28.0-30.0% NH_3 by weight) were purchased from Alfa Aesar (Ward Hill, USA). The Gd_2O_3, Yb_2O_3 and Er_2O_3 were reacted with excess hydrochloric acid to form the rare-earth chloride compounds, respectively. Then, the rare-earth chloride compounds were dried by solvent evaporation, and redispersed in water to yield the $GdCl_3$ (1.6 M), $YbCl_3$ (0.6 M) and $ErCl_3$ (0.1 M) aqueous stocking solutions, respectively. N,N'-Methylenebisacrylamide solution (bis-acrylamide, 2% (w/v) in H_2O), 3-(trimethoxysilyl) propyl methacrylate (≥ 98%), acrylic acid (99%), bovine serum albumin (BSA), 1-octadecene (ODE, 90%), oleic acid (OA, 90%), Triton X-100 and tetraethyl orthosilicate (TEOS, 99.999%) were purchased from Sigma-Aldrich Chemical Co. Ltd. (St. Louis, MO, USA). Carboxyethylsilanetriol (CTES, sodium salt, 25% in water) was purchased from Leon Technology Co. Ltd. (Beijing, China). Benzophenone (BP), *n*-hexane and cylcohexane were supplied by Sinopharm Chemical Reagent Co. Ltd. (Beijing, China). Dulbecco's modified Eagle's medium (DMEM) and fetal bovine serum (FBS), ammonium fluoride (NH_4F, 98%) and 3-(4,5-dimethylthiazol-2-yl)-2,5-diphenyltetrazolium bromide (MTT) were purchased from Beijing Dingguo Biotechnology Ltd. (Beijing, China). Leibovitz's L-15 culture medium and McCoy's 5A culture medium were purchased from Jiangsu KenGEN BioTECH Corp., Ltd. (Jiangsu, China). 27 lectins (see **Table S1** for more details) were products of Vector Laboratory Ltd. (Burlingame, CA, USA). Polytetrafluoroethylene (PTFE) grid, PTFE masker paste tool and glass microscope slides were obtained from CapitalBio Ltd. (Beijing, China). Other reagents (analytical grade) were purchased from Beijing Chemical Reagents Company (Beijing, China). All reagents were

used as received without further purification. Milli-Q water (18.2 MΩ cm) was used in all experiments.

Characterization: Transmission electron microscope (TEM) and high-magnification TEM (HRTEM) micrographs were performed on a FEI Tecnai G2 S-Twin TEM (FEI Co., USA) with a field emission gun operating at 200 kV. Scanning electron microscope (SEM) micrographs were performed on filed emitted SEM (XL 30 ESEM FEG, FEI Co., USA). Atomic force microscope (AFM) was carried out on BioScope Resolve AFM (Bruker Nano Surfaces, Santa Barbara, CA, USA) with ScanAsyst-Air mode using Air probes (tip radius, 2-12 nm and silicon nitride cantilever; spring constant, 0.4 N m^{-1}). All images were obtained with an 1 Hz scan rate. AFM images were analyzed quantitatively by means of Nanoscope Analysis1.8 (Bruker Nano Surfaces, Santa Barbara, CA, USA). The powder X-ray diffraction analysis (XRD) was carried out on a D8 ADVANCE diffractometer (Bruker Co., Germany) using Cu Ka (0.15406 nm) radiation. The X-ray photoelectron spectroscopy (XPS) was recorded on a VGESCALAB MKII spectrometer (VG Scientific Ltd., UK). All dynamic light scattering (DLS) and zeta potential distribution measurements were carried out on a Zetasizer Nano ZS (Malvern Instruments Ltd., UK). The elemental analysis was performed by an ICAP 6000 ICP-OES system (Thermo Scientific, Waltham, MA, USA). Fourier transform infrared (FTIR) spectroscopic analysis was performed on a Bruker Vertex 70 spectrometer. The upconversion luminescence (UCL) spectra were recorded with a 980 nm laser from an optical parametric oscillator (OPO) (Continuum Sunlite) as the excitation source. The UCL imaging was performed with a reconstructive Nikon Ti-S fluorescent microscope (Nikon, Tokyo, Japan) equipped with a CW NIR laser at 980 nm. T_1-weighted MR images of UCNP@SiO_2-COOH and UCNP@SiO_2-UEA-I aqueous solutions with various concentrations were acquired using Siemens Prisma 3.0 T MR scanner (Erlangen, Germany) with gradient strength up to 80 Mt m^{-1}. Imaging parameters were as follows: repetition time (TR), 12000ms; seven inversion recovery times (TI = 20, 40, 80, 160, 320, 640, 1280 and 2560 ms). CT images of

UCNP@SiO_2-COOH and UCNP@SiO_2-UEA-I aqueous solutions with various concentrations were acquired using a 64-detector row CT unit (General Electric, Milwaukee, WI). Imaging parameters were as follows: thickness, 0.6 mm; pitch, 0.99; 120 kVp, 300 mA; field of view, 75.5 mm; gantry rotation time, 0.5 s, and table speed, 15.9 mm s^{-1}.

2. Additional Figures S1-S16

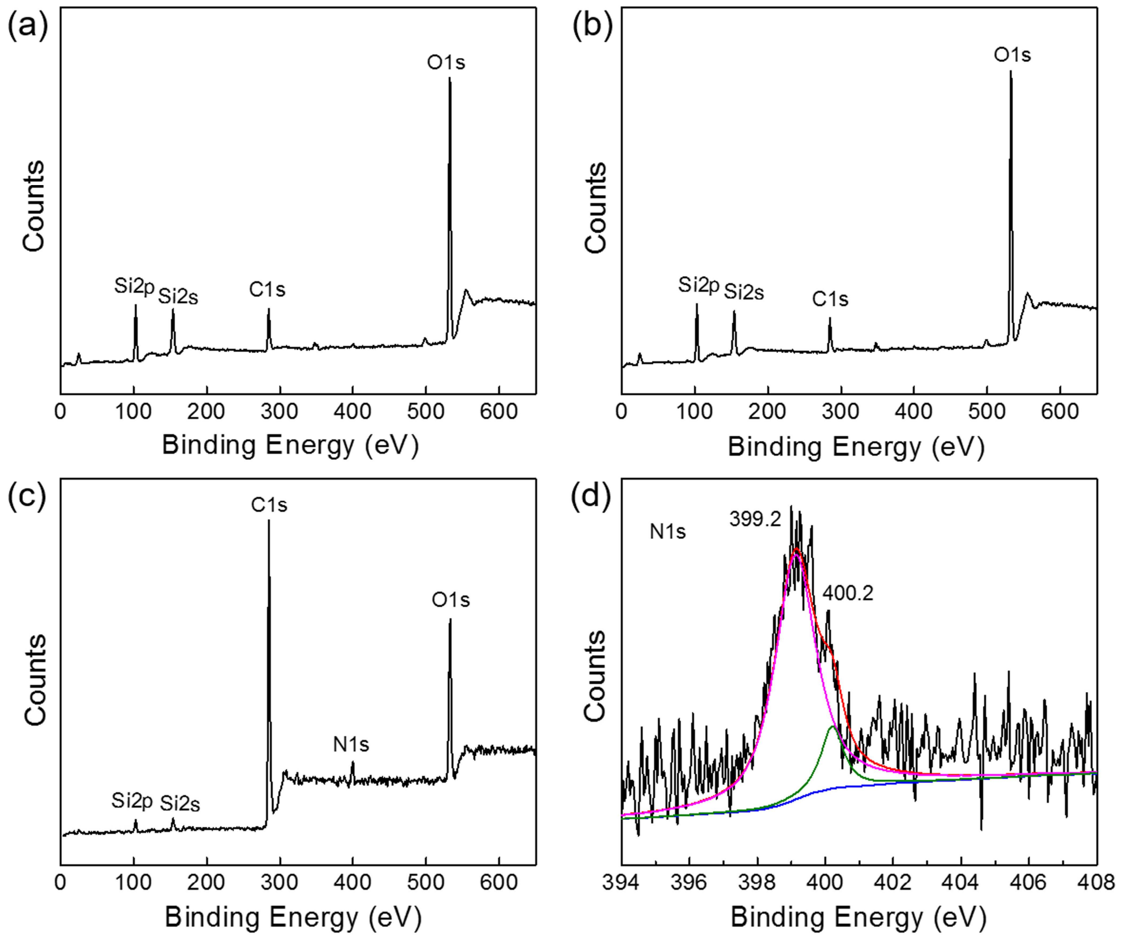

Figure S1. XPS wide scan of the glass slides: (a) hydroxylated slide, (b) silanization solution and BP modified glass slide, (c, d) polyacrylamide hydrogel modified glass slide and the detailed N1s.

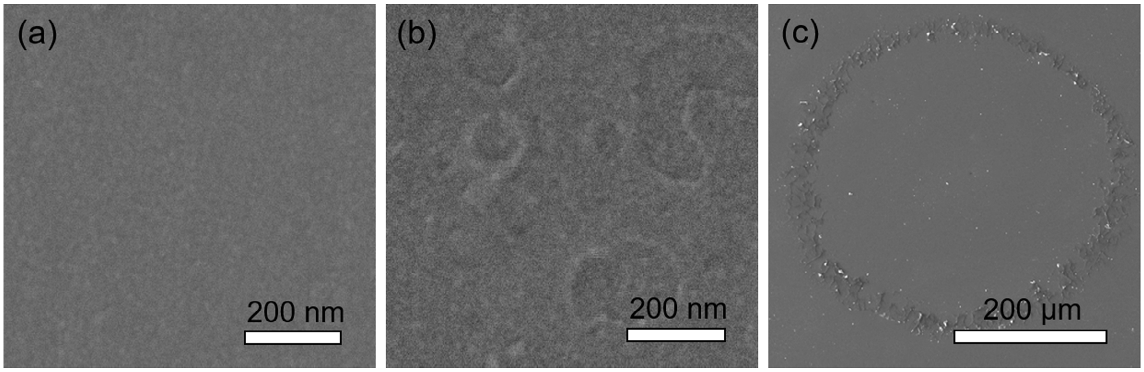

Figure S2. SEM micrographs of the glass slides before (a) and after (b, c) modification of polyacrylamide hydrogel.

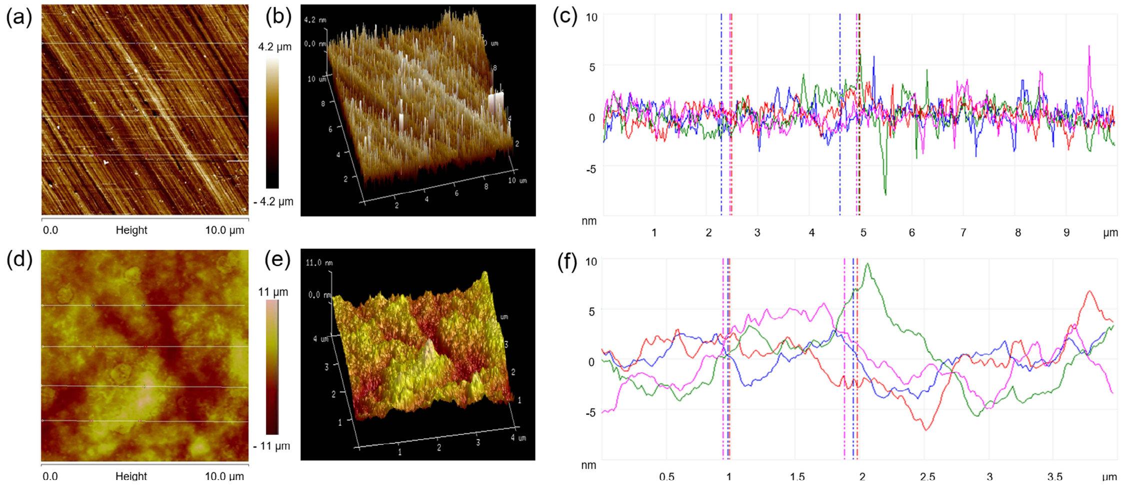

Figure S3. AFM micrographs and corresponding three-dimensional images as well as the cross-section profile along the white line in (a) and (d) before (a-c) and after (d-f) modification of polyacrylamide hydrogel.

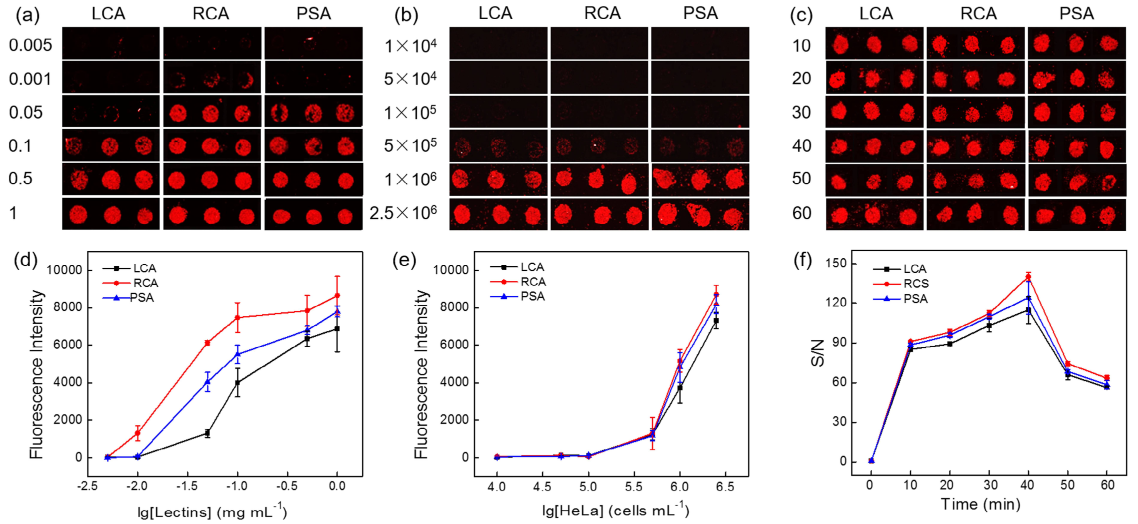

Figure S4. Effects of the lectin concentrations (a, d), cells density (b, e) and incubation time (c, f) on the lectin-cell binding capacity.

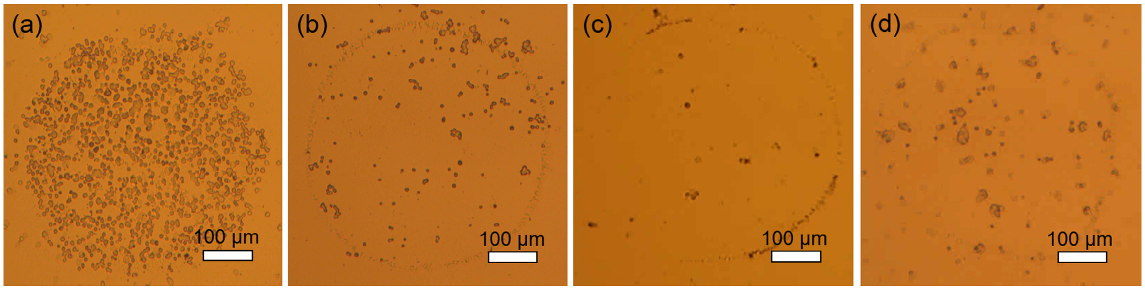

Figure S5. The microscope images of SW480 (a), SW620 (b), HCT116 (c) and NCM460 (d) captured by immobilized UEA-I on PAAM hydrogel spot, respectively.

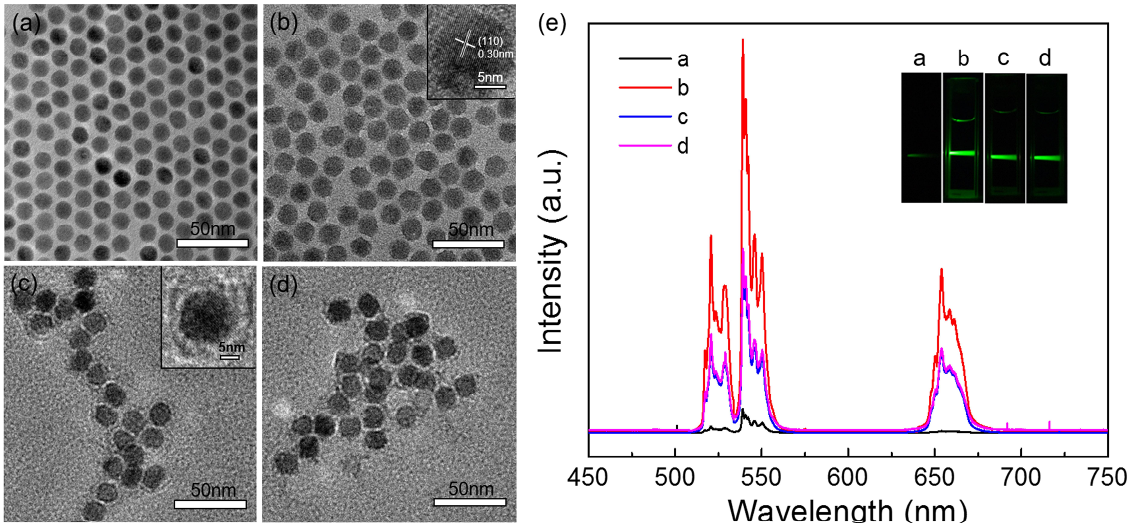

Figure S6. TEM micrographs (a to d) and UCL spectra (e) of $NaGdF_4$:Yb^{3+}, Er^{3+} UCNPs (a), $NaGdF_4$:Yb^{3+}, Er^{3+}@$NaGdF_4$ UCNPs (b), UCNP@SiO_2-COOH (c) and UCNP@SiO_2-UEA-I (d). Insets of (b) and (c) are corresponding HRTEM micrographs of the UCNPs while inset of (e) is the corresponding digital photographs of luminescence of UCNPs.

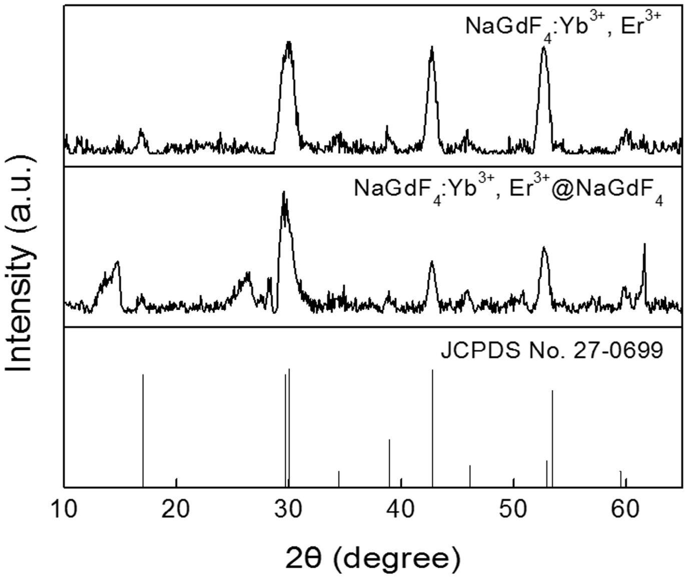

Figure S7. The power XRD patterns of $NaGdF_4:Yb^{3+}$, Er^{3+} UCNPs, and $NaGdF_4:Yb^{3+}$, $Er^{3+}@NaGdF_4$ UCNPs, respectively.

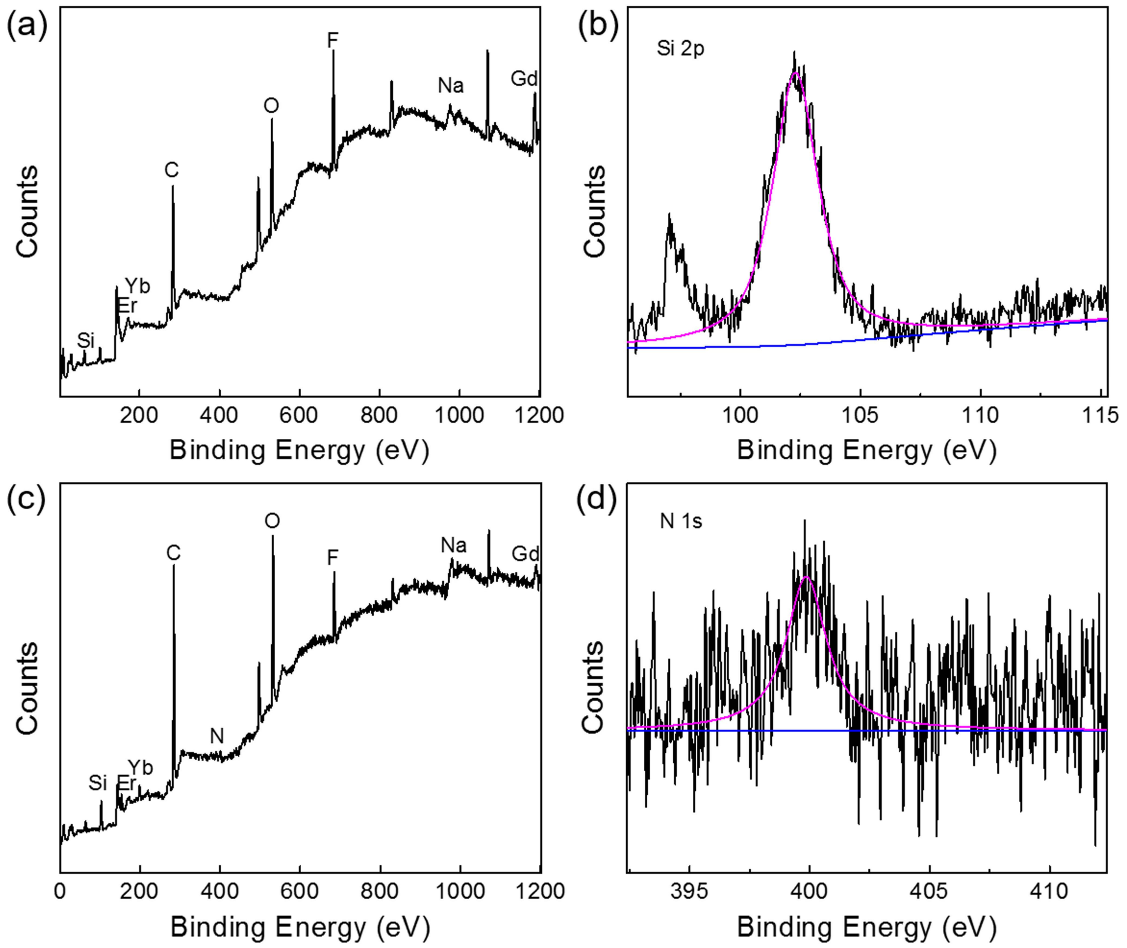

Figure S8. XPS spectra of UCNP@SiO_2-COOH (a, b) and UCNP@SiO_2-UEA-I (c, d), respectively.

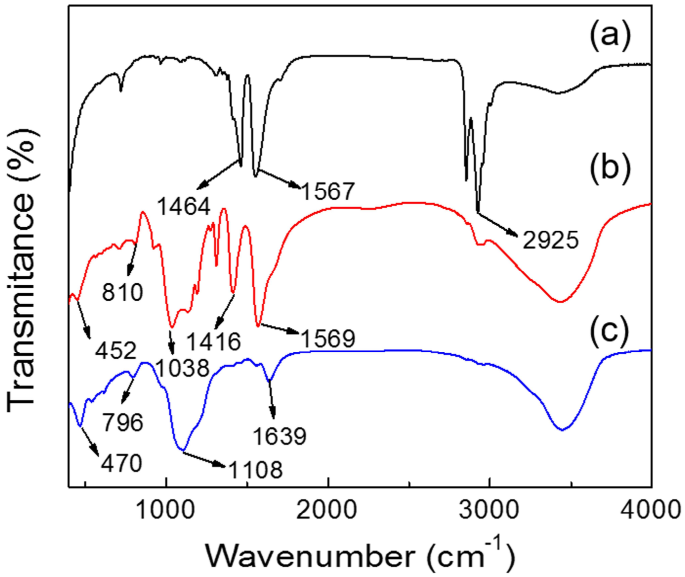

Figure S9. FTIR spectra of (a) oleic acid stabilized $NaGdF_4$: Yb^{3+}, Er^{3+}@$NaGdF_4$ UCNPs, (b) UCNP@SiO_2-COOH and (c) UCNP@SiO_2-UEA-I, respectively.

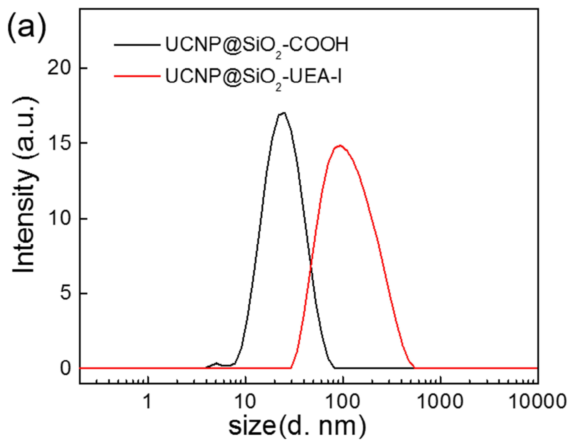

Figure S10. Hydrodynamic size distribution (a) and zeta potentials (b) of UCNP@SiO$_2$-COOH and UCNP@SiO$_2$-UEA-I dispersed in PBS buffer (pH 7.4), respectively.

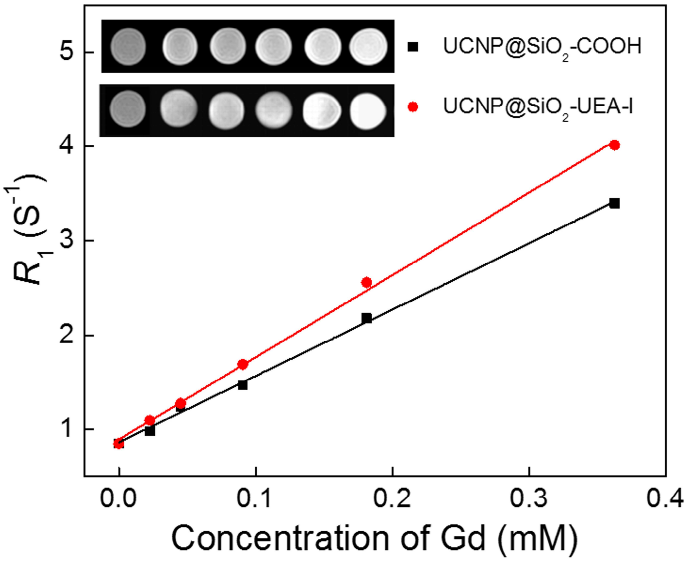

Figure S11. T_1-weighted MR images and relaxation rate (R_1) of aqueous solutions of UCNP@SiO_2-COOH and UCNP@SiO_2-UEA-I as a function of the molar concentration of Gd in the solution, respectively.

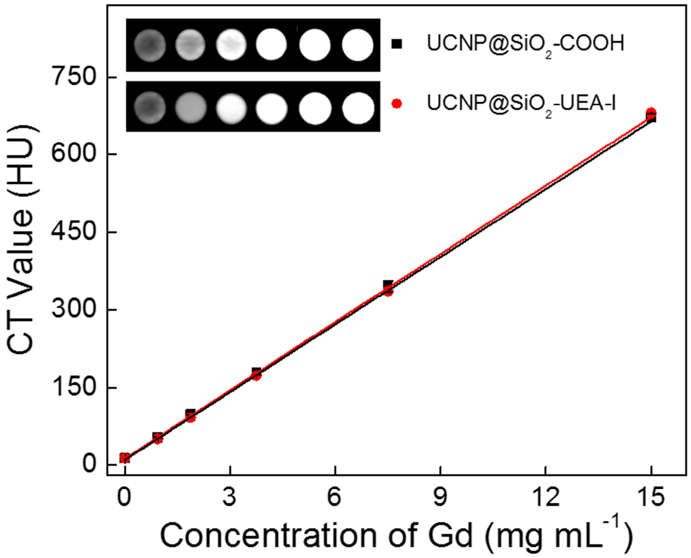

Figure S12. CT images and HU value of UCNP@SiO_2-COOH and UCNP@SiO_2-UEA-I aqueous solutions as a function of the mass concentration of Gd in the solution, respectively.

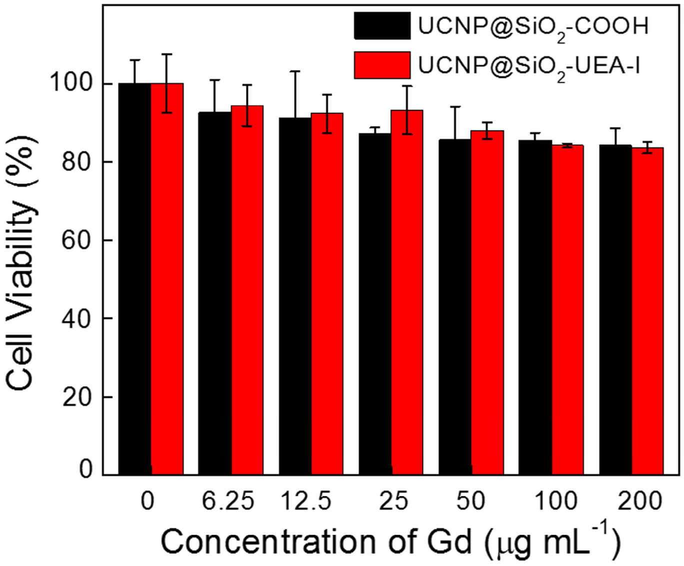

Figure S13. In vitro cell viabilities of SW480 cells incubated with various concentrations (0, 6.25, 12.5, 25, 50, 100 and 200 μg mL^{-1}) of UCNP@SiO_2-COOH and UCNP@SiO_2-UEA-I for 24 h, respectively. The error bars mean standard deviations (n = 3).

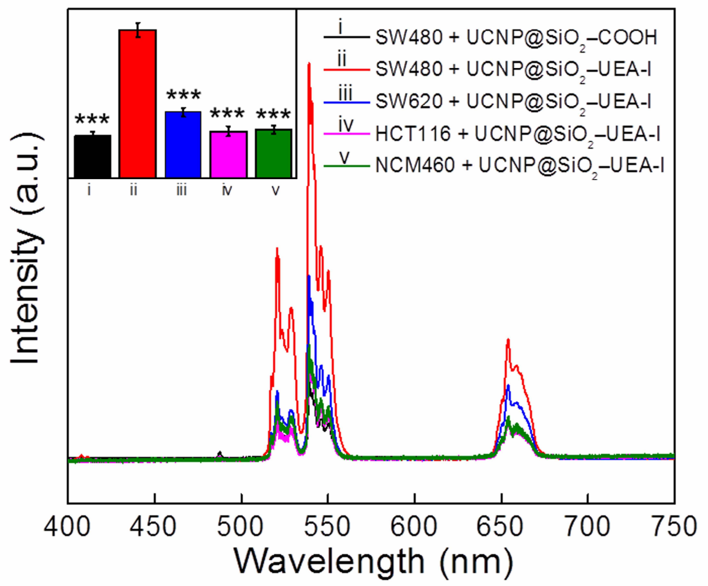

Figure S14. UCL spectra of UCNP@SiO_2-UEA-I stained SW480 cells, SW620 cells, HCT-116 cells and NCM460 cells, and UCNP@SiO_2-COOH stained SW480 cells, respectively. Inset is the corresponding UCL intensities at 539 nm of the NPs stained cells. The error bars are standard deviations (n = 3. The significance of the data is analyzed according to one-sided paired Student's *t*-test: ***P < 0.001).

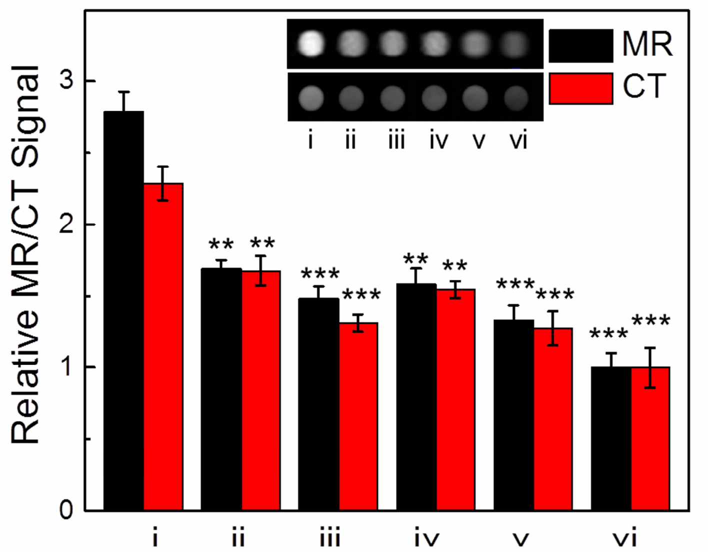

Figure S15. MR/CT imaging and relative MR/CT signals of UCNP@SiO2-UEA-I stained SW480 cells (i), SW620 cells (ii), HCT116 cells (iii) and NCM460 cells (iv), UCNP@SiO2-COOH stained SW480 cells (v), and unstained SW480 cells (vi), respectively. The error bars are standard deviations (n = 3. The significance of the data is analyzed according to one-sided paired Student's *t*-test: $^{**}P < 0.01$ and $^{***}P < 0.001$).

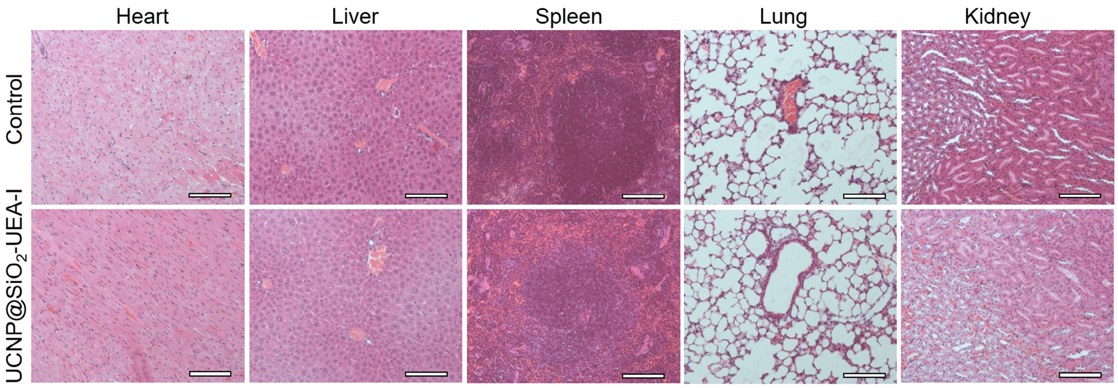

Figure S16. Histological changes of healthy mouse without injection of UCNP@SiO_2-UEA-I (a-e) and the mouse after 30 days post-injection of a single dose of UCNP@SiO_2-UEA-I (Gd content: 10 mg kg^{-1}) in 200 μL NaCl solution (0.9 wt %) (f-j), respectively. Scale bars are 200 μm.

3. Additional Tables S1-S5

Table S1. Lectins and their special binding of saccharide groups.

Number	Lectin	Common Abbreviation	Binding Specificity[a,b]
1	Bauhinia Purpurea Lectin	BPL	Galβ3GalNAc
2	Uelx Europaeus Agglutinin I	UEA-I	αFuc
3	Lotus Tetragonolobus Lectin	LTL	αFuc
4	Griffonia Simplicifolia Lectin I	GSL-I	αGal, αGalNAc
5	Griffonia Simplicifolia Lectin II	GSL-II	α or βGlcNAc
6	Maackia Amurensis Lectin I	MAL-I	Galβ4GlcNAc
7	Maackia Amurensis Lectin II	MAL-II	α-2,3 sialic acid
8	Euonymus Europaeus Lectin	EEL	Galα3Gal
9	Narcissus Pseudonarcissus (Daffodil) Lectin	NPL	αMan
10	Datura Stramonium Lectin	DSL	(GlcNAc)2-4
11	Amaranthus Caudatus Lectin	ACL	Galβ3GalNAc
12	Hippeastrum Hybid (Amaryllis) Lectin	HHL	αMan
13	Pisum Sativum Agglutinin	PSA	αMan,αGlc
14	Lens Culinaris Agglutinin	LCA	αMan,αGlc
15	Soybean Agglutinin	SBA	α>βGalNAc
16	Elderberry Bark Lectin	EBL	Neu5Acα6Gal/GalNAc
17	Ricinus Communis Agglutinin I	RCA 120	Galβ(1,4)GlcNAcβ1
18	Solanum Tuberosum (Potato) Lectin	STL	(GlcNAc)2-4
19	Dolichos Biflorus Agglutinin	DBA	αGalNAc
20	Jacalin	Jacalin	Galβ3GalNAc
21	Erythrina Cristagalli Lectin	ECL	Galβ4GlcNAc
22	Aleuria Aurantia Lectin	AAL	Fucα6GlcNAc
23	Concanavalin A (Con A), Unconjugated	ConA	αMan, αGlc
24	Wheat Germ Agglutinin	WGA	β-GlcNAc, sialic acid, GalNAc
25	Peanut Agglutinin	PNA	Galβ3GalNAc
26	Galanthus Nivalis Lectin	GNL	αMan
27	Phaseolus Vulgaris Agglutinin	PHA-E+L	Galβ4GlcNAcβ2

[a)]The binding specificity is obtained from manufacture's introduction.
[b)]Sugar Abbreviations:

Fuc → L-Fucose　　GlcNAc → N-Acetylglucosamine
Gal → D-Galactose　　GalNAc → N-Acetygalactosamine
Glc → D-Glucose

Table S2. The layout of the lectin microarray with 27 lectins. Each lectin was spotted with three duplicates.

BPL	GSL II	LTL	AAL
PNA	HHL	WGA	Jacalin
DBA	NPL	GSL I	MAL I
UEA-I	ECL	Con A	PHA
LCA	MAL II	PSA	RCA
ACL	STL	GNL	SBA
DSL	EBL	EEL	Control

Table S3. T_1 signal intensities on tumor of SW480 tumor- and HCT116 tumor-bearing nude mice after intravenous injection with UCNP@SiO_2-COOH and UCNP@SiO_2-UEA-I at different timed intervals, respectively (the 0 h means pre-injection).

Signal intensity	0 h	1 h	2 h	24 h
SW480+UCNP@SiO_2-COOH	892	1081	1156	1270
SW480+UCNP@SiO_2-UEA-I	735	1083	1777	1915
HCT116+UCNP@SiO_2-COOH	687	807	881	984
HCT116+UCNP@SiO_2-UEA-I	1275	1522	1611	1876

Table S4. CT values on tumor of SW480 tumor- and HCT116 tumor-bearing nude mice after intravenous injection with UCNP@SiO_2-COOH and UCNP@SiO_2-UEA-I at different timed intervals, respectively (the 0 h means pre-injection).

Signal intensity	0 h	1 h	2 h	24 h
SW480+UCNP@SiO_2-COOH	32	39	44	48
SW480+UCNP@SiO_2-UEA-I	32	47	58	92
HCT116+UCNP@SiO_2-COOH	30	39	41	43
HCT116+UCNP@SiO_2-UEA-I	26	33	37	41

Table S5. Results of blood biochemical assays.

Parament	Units	Control	Treatment
WBC	10^9 L^{-1}	0.81	0.59
LYMPH	10^9 L^{-1}	0.43	0.53
RBC	10^{12} L^{-1}	6.63	8.49
HGB	g L^{-1}	126.00	134.00
HCT	%	31.70	38.60
MCV	fL	47.80	45.50
MCH	pg	19.00	15.80
MCHC	g L^{-1}	397.00	347.00
PLT	10^9 L^{-1}	170.00	186.00
